# Advances in Hole Transport Materials for Layered Casting Solar Cells

**DOI:** 10.3390/polym15224443

**Published:** 2023-11-17

**Authors:** Vu Khac Hoang Bui, Thang Phan Nguyen

**Affiliations:** 1 Department of Environment and Energy, Sejong University, Seoul 05006, Republic of Korea; buikhachoangvu@sejong.ac.kr; 2 Department of Chemical and Biological Engineering, Gachon University, Seongnam-si 13120, Gyeonggi-do, Republic of Korea

**Keywords:** polymer solar cells, perovskite solar cell, hole transport layer, inorganic HTL, organic HTL

## Abstract

Huge energy consumption and running out of fossil fuels has led to the advancement of renewable sources of power, including solar, wind, and tide. Among them, solar cells have been well developed with the significant achievement of silicon solar panels, which are popularly used as windows, rooftops, public lights, etc. In order to advance the application of solar cells, a flexible type is highly required, such as layered casting solar cells (LCSCs). Organic solar cells (OSCs), perovskite solar cells (PSCs), or dye-sensitive solar cells (DSSCs) are promising LCSCs for broadening the application of solar energy to many types of surfaces. LCSCs would be cost-effective, enable large-scale production, are highly efficient, and stable. Each layer of an LCSC is important for building the complete structure of a solar cell. Within the cell structure (active material, charge carrier transport layer, electrodes), hole transport layers (HTLs) play an important role in transporting holes to the anode. Recently, diverse HTLs from inorganic, organic, and organometallic materials have emerged to have a great impact on the stability, lifetime, and performance of OSC, PSC, or DSSC devices. This review summarizes the recent advances in the development of inorganic, organic, and organometallic HTLs for solar cells. Perspectives and challenges for HTL development and improvement are also highlighted.

## 1. Introduction

Perovskite solar cells (PSCs), dye-sensitive solar cells (DSSCs), and polymer solar cells (PSCs) are examples of layered casting solar cells (LCSCs). PSCs are a particular kind of solar cell that use lead- or tin halide-based materials with perovskite structures as their light-harvesting active layer [1,2]. The power conversion efficiency (PCE) of lab-scale PSCs has grown from 3.8% in 2009 to 26.1% in 2023 for single-junction structures and 29.8% for silicon-based tandem structures [1,3,4,5,6]. Perovskite solar cells have drawn a lot of research attention because of their promise for high efficiency, affordable manufacturing, and compatibility with a variety of device topologies [7,8]. Ongoing research is focused on addressing challenges related to stability, scalability, and toxicity concerns associated with lead-based perovskites [7,8]. Organic photovoltaics (OPVs), commonly referred to as organic solar cells (OSC), are a subclass of LCSCs for which the active layer for converting sunlight into energy is made of organic components. OSCs offer advantages such as flexibility, lightweight design, and the potential for low-cost fabrication through solution-based processes [9,10,11]. The performance and efficiency of OSCs depend on the choice of donor and acceptor materials, as well as the morphology of the active layer, which can be optimized through processing techniques. While they typically have lower efficiency compared to inorganic solar cells, ongoing research aims to improve their performance and commercial viability [9,10,11]. DSSCs consist of a porous semiconductor film (usually titanium dioxide, TiO_2_) coated with a light-absorbing dye, an electrolyte solution, and a counter electrode. The dye absorbs photons and generates excited electrons, which are introduced into the semiconductor material. These electrons are then collected at the electrode, creating an electric current [12,13]. In general, both perovskite and organic solar cells have their own unique advantages and challenges. Perovskites solar cells have shown remarkable efficiency improvements but need further development in terms of stability. Meanwhile, OGCs have advantages in terms of flexibility and lightweight design, but efficiency and stability improvements are ongoing research goals. DSSCs, while having lower efficiency compared to other types of solar cells, offer advantages in low light or under artificial conditions [12,13].

LCSCs have various advantages compared to conventional solar cells. LCSCs can collect more sunlight by using different materials with different band gaps and absorption spectra. LCSCs can be more efficient by reducing thermalization losses, increasing open-circuit voltage, and improving charge separation. By using cheaper or more abundant materials, such as organic polymers or perovskites, and simpler fabrication methods, such as solution processing or hot casting, LCSCs have lower fabrication costs [12,14,15]. However, in general, LCSCs still maintain some limitations. LCSCs have poor stability due to the degradation of materials, interfacial defects, moisture sensitivity, or thermal stress. It is difficult to optimize LCSCs due to the interplay of different layers, the trade-off between current and voltage, and the compatibility of different materials. Lastly, the environmental issue remains due to the application of toxic or scarce materials (lead or indium) and the generation of waste or emissions during production [12,14,15].

Charge separation in solar cells relies on the generation of electron–hole pairs upon photo absorption, the creation of an electric field within the semiconductor material, and the motion of charged carriers to their respective region [12,16]. The separated electrons and holes travel through an external circuit to power electrical devices or charge a battery [12,16]. In many LCSCs, including perovskite solar cells and organic solar cells, a hole transport layer (HTL) is utilized to speed up the extraction and transportation of positive charge carriers (holes) from the active layer to the anode [8,14,17]. The HTL also serves as a barrier to stop electron leakage to the anode and as a layer of defense to shield the active layer from oxygen and moisture [8,14,17]. The HTL can be made of inorganic and organic materials, depending on the compatibility, cost, stability, and performance of the solar cells [14,17]. An ideal material for the HTL should have high hole mobility, excess holes, a relatively narrow bandgap, low defect density, good stability, and compatibility with other layers [8,18,19,20]. In this review, we focus on the recent progress of inorganic and organic HTLs in LCSCs, their advantages and disadvantages, and discuss the remaining challenges of each type of HTL that should be overcome in future studies.

## 2. Solar Cell Factors

**Figure 1:**
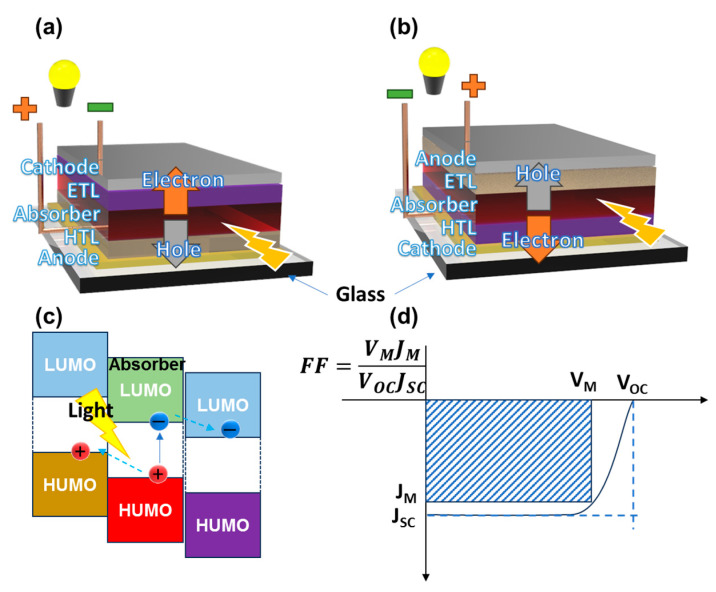
(**a**) Conventional and (**b**) inverted structures of an organic solar cell; (**c**) band gap alignment of active material with electron/hole transport; (**d**) current density (J)–voltage (V) curves of the solar cell with the inset of the fill factor equation.

A typical OSC is made up of an absorber, an electron transport layer (ETL), a hole transport layer (HTL), and anode and cathode electrodes [16]. One of the two electrodes is a substrate-compact transparent conducting oxide (TCO) electrode, which provides the conductive layer and allows most light to pass through. The TCO generally consists of indium tin oxide (ITO) or FTO (fluorine-doped tin oxide), the transparency of which is up to 93% [21]. An absorber is an organic/hybrid/inorganic semiconductor that can highly absorb light. It is also called an active material. In organic semiconductors, the lowest unoccupied molecular orbital (LUMO) and the highest occupied molecular orbital (HOMO) are the energy bands, which are similar to the conduction band (CB) and valence band (VB) of inorganic semiconductors, relating to electrons and holes as charge carriers, respectively. When light strikes the solar cell, the active material will absorb photons and form excitons (electron–hole couples) in the HOMO. The electrons and holes will immediately be separated to the LUMO and HUMO, respectively. Then, the ETL and HTL will transport charge carriers to the two electrodes, creating a different voltage potential for a solar cell. If the TCO is on the anode side, it is a conventional structure, and if it is on the cathode side, it is an inverted structure, as shown in Figure 1a. Therefore, the hole can be collected from the front or back side of the cell, depending on the conventional or inverted structure, respectively. This structure will be determined by the casting layer technique or the properties of each layer. When irradiating light to an OSC, photoelectric conversion occurs, which includes three main processes: (I) light absorption and creation of excitons, (II) separation of excitons to electrons and holes, and (III) selective collection of charge carriers. An ideal OSC should have a good absorber, and the ETL and HTL should provide quick carrier transport to prevent the recombination of electrons and holes. In energy band expression, the layers of the OSC must follow the band alignment to optimize the carrier transport process, which is described in Figure 1b. Thus, the HTL is an electron-blocking material in addition to being a hole transport material. The ETL transports electrons and blocks holes. Later, more layer structures are established using separating hole/electron-blocking layers.

To evaluate a solar cell’s performance, there are some major factors, including the fill factor (FF), power conversion efficiency (PCE), open-circuit voltage ($V_{OC}$), and short-circuit current density ($J_{SC}$). The PCE is calculated using the following equation:

$$PCE=\frac{V_{OC}\times J_{SC}\times FF}{P_{I}}$$

where $P_{I}$ is the power density of incident light; $V_{OC}$ is the highest voltage that the cell can supply; and $J_{SC}$ is the highest current density for the output. 

FF is the ratio of maximum power output to maximum obtainable power output ($FF=P_{max}/(V_{OC}\times J_{SC})$), as shown in Figure 1c. Thus, FF indicates the quality of a solar cell, which is the obtainability of high current density and high voltage or output power. To obtain a standard measurement, solar cells are generally irradiated by a solar simulator light source with a power of 1000 W m^−2^, and its light spectrum is equivalent to the light of AM 1.5 spectrum [22]. The PCE indicates how many percentages of incident light can be converted to an electric signal for output. The PCE depends much on the absorber and the efficiency of the charge transfer process by the ETL and HTL. A conventional cell with an ITO/PEDOT:PSS/P3HT:PC_61_BM/PC61BM/LiF/Al structure can exhibit a PCE of 3.0–4.0%. If PC_61_BM is replaced by PC_71_BM, the PCE can be 7.0–8.0%, or by PTB7:PC_71_BM, the PCE can be 7.0–9.0%, etc. [23]. Therefore, when investigating the electron transport material (ETM) and hole transport material (HTM) in a solar cell, reference cells with or without a traditional ETM, such as PCBM, or HTM, such as PEDOT:PSS, should be prepared at the same time. In this study, the effect of various HTMs on solar cell performance will be revealed. The energy level, work function design, and surface engineering technique play important roles in achieving the reproducibility of organic layered solar cells.

## 3. Organic Hole Transport Layer (OHTL)

**Figure 2:**
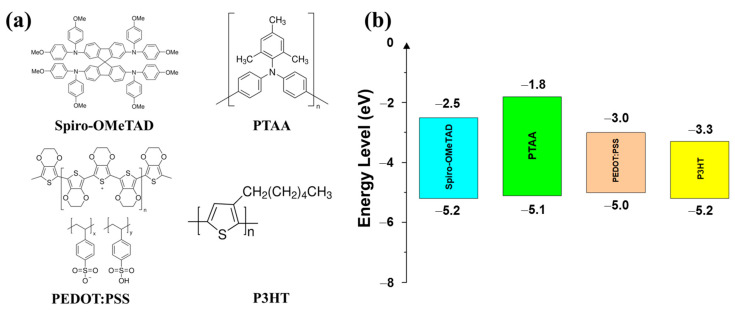
(**a**) Common organic hole transport materials (OHTMs) and (**b**) their energy levels. Reproduced with permission from ref. [14]. Copyright 2021, Springer Nature.

The organic hole transport layer (OHTL) is a layer of organic material that is used in LCSCs to extract and transport the holes (positive charges) from the active layer to the electrode [16,17,24]. The performance and stability of solar cells can be increased via the OHTL. To stop the transfer of electrons (negative charges) to the anode, the OHTL functions as an energy barrier [17,24]. The OHTL isolates the moisture in the air, which might deteriorate the active layer, by separating it from the anode [17,24]. OHTLs increase the solar cell’s open-circuit voltage while decreasing charge recombination [16,17]. High charge carrier mobility and conductivity are ideal requirements for OHTLs. The HOMO and quasi-Fermi levels of OHTLs should be suitable for reliable hole transfer in a pinhole-free morphology, while OHTLs should also have a high LUMO for efficient electron shielding, thermal, moisture, UV, and chemical resistance, and good morphological contact between the perovskite and the HTL. HTLs should also be created using inexpensive and readily available materials, inexpensive solution-based fabrication techniques that do not harm other solar cell components, and easily reproducible synthesis [14]. Remarkable organic materials that are applied as OHTLs are poly(3,4-ethylenedioxythiophene) (PEDOT), N,N’-bis(3-methylphenyl)-N,N’-diphenylbenzidine (spiro-MeOTAD), poly(3-hexylthiopene) (P3HT), and poly(triaryl amine) (PTAA) (Figure 2a). The energy levels of these materials are presented in Figure 2b.

### 3.1. Spiro-OMeTAD

The most prevalent HTM in LCSCs is spiro-OMeTAD [14]. Spiro-OMeTAD was used for the first time in PSCs in 2012, displaying an excellent efficiency of 9.7% and significantly better stability than liquid junction PSCs [25]. However, high performance of solar cells can be achieved when spiro-OMeTAD is combined with p-dopants such as lithium bis(trifluoromethanesulfonimidate) (Li-TFSI), 4-*tert* butyloyridine (tBP), and tris[2-(1*H*-pyrazol-1-yl)-4-*tert*-butylpyridine]cobalt(III)tris(bis(tri-fluoromethylsulfonyl)imide)] [26,27]. By doping with p-dopants, spiro-OMeTAD is converted to spiro-OMeTAD^+^ [28]. Additionally, this is 420 meV deeper in its HOMO, which may result in improved energy alignment at spiro-OMeTAD/perovskite contact [29]. Spiro-OMeTAD has a high hole mobility (μ_h_) of 2 × 10^−4^ cm^2^⋅V^−1^⋅s^−1^ [26], allowing it to efficiently transport positive charges within the perovskite layer to the electrode and minimize charge recombination losses, thus enhancing device performance. Spiro-OMeTAD possesses suitable energy level alignment, allowing it to selectively transport holes while preventing the transport of electrons. At perovskite contact, spiro-OMeTAD displays a LUMO of −1.5 eV and a HOMO of −5.0 eV (0.1 eV). A number of distinct perovskite conduction bands (CB) are more than 2 eV above the LUMO of spiro-OMeTAD, which suggests strong blocking properties [30]. This guarantees efficient and loss-free extraction of charge carriers from the perovskite layer. Spiro-OMeTAD has shown good chemical and thermal stability, which is important for maintaining the long-term performance of perovskite solar cells. Spiro-OMeTAD is compatible with the deposition techniques commonly used for perovskite fabrication, such as solution processing. On top of the perovskite material, a smooth and homogeneous layer of spiro-OMeTAD can be created by using a suitable solvent, such as chloroform [31,32]. The structure of spiro-OMeTAD allows for efficient hole extraction at the interface between the perovskite absorber layer and the HTM layer, reducing charge recombination and enhancing device efficiency [18]. Lastly, spiro-OMeTAD is commercially available and relatively easy to synthesize, making it accessible for researchers and manufacturers.

**Figure 3:**
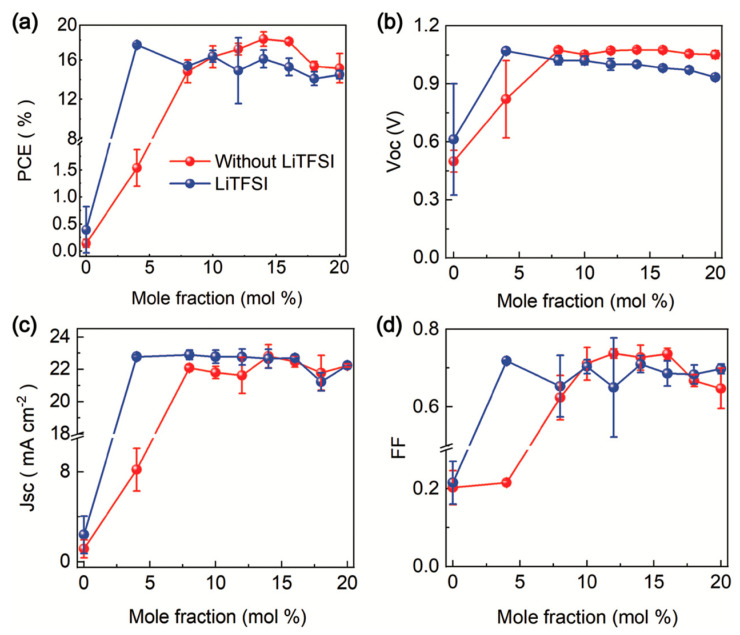
Performance of PSCs based on spiro-OMeTAD(TFSI)_2_ in the absence (red) and existence (blue) of LiTFSI: (**a**) PCE, (**b**) $V_{OC}$, (**c**) $J_{SC}$, (**d**) FF. Reprinted with permission from ref. [38] Copyright 2019, Wiley-CH.

Spiro-OMeTAD, despite its popularity as an HTM in PSCs, has several limitations that researchers are actively working to address. Spiro-OMeTAD is remarkable costly at a staggering 29.36 USD per milliliter, and the synthesis of spiro-OMeTAD can be quite intricate, involving multiple steps that necessitate the use of expensive materials, such as palladium (Pd) catalysts [33,34]. Additionally, its commercial availability can lead to higher costs compared to other materials, potentially affecting the scalability of perovskite solar cell manufacturing. Due to exposure to air, moisture, and light, spiro-OMeTAD can deteriorate over time. This can lead to a decrease in device performance and efficiency, particularly in long-term outdoor applications. For example, the dopants Li-TFSI and tBP easily break down spiro-OMeTAD and perovskite films, which makes the PCE less stable over time. Recently, Liu et al. (2023) introduced 1-dodecanethiol (DDT), an alkylthiol additive, in spiro-OMeTAD. This integration made the structure of the HTM more resistant to heat, moisture, and light stress, shortened the time it takes to dope, and reduced the amount of Li-TFSI_2_ that builds up at the interfaces. The devices based on spiro-OMeTAD stabilized with DDT exhibited a PCE of 23.1%. The units could maintain 90% of their peak performance for 1,000 h of continuous illumination [35]. The moisture resistance of spiro-OMeTAD-based solar cells can also be enhanced by the replacement of Li-TFSI with more hydrophobic additives such as Zn-TFSI_2_, Mg-TFSI_2_, and Ca-TFSI_2_ [36,37]. There are also some efforts to produce spiro-OMeTAD-based PSCs without the use of Li-TFSI. The LiTFSI-free spiro-OMeTAD by Tan et al. (2019) could achieve a PCE of 19.3% (Figure 3) [38]. While spiro-OMeTAD has been successful in achieving suitable energy level alignment for hole transport, it is not a perfect match for all perovskite compositions. Tailoring energy level alignment to different perovskite materials can be a challenge. At elevated temperatures, the performance of spiro-OMeTAD-based devices is decreased due to the significantly lower glass transition temperature of oxidized spiro-OMeTAD^+^ [39,40,41]. In addition, weak contacts at the spiro-OMeTAD/perovskite interface and reinforcement of the film brought on by a buildup of additives at the interface cause poor adhesion [42]. By including a polyethyleneimine (PEI) interlayer between spiro-OMeTAD and perovskite, this restriction can be bypassed [43]. Additionally, under continuous illumination, photodegradation of the chemical interaction between spiro-OMeTAD and Au at the interface is seen [44]. In addition, spiro-OMeTAD is a poor barrier for Au migration into the perovskite [18]. To prevent electrode diffusion and boost stability, a bilayer Cu-Ag electrode has been employed [45]. Spiro-OMeTAD contains heavy metals, which can raise environmental and health concerns. The use of toxic or environmentally unfriendly materials is an ongoing challenge for sustainable solar cell technologies. Spiro-OMeTAD itself does not absorb light in the visible spectrum, which means it does not contribute to light absorption in the device. This can lead to suboptimal utilization of incident sunlight. In thick perovskite layers, it can be hard for spiro-OMeTAD to pull out charges that are made deep within the perovskite layer. This could cause charges to build up and energy to be lost.

Spiro-OMeTAD comprises a spirobifluorene core bound to four bis(methoxyphenyl)amines. The derivatives of bis(methoxyphenyl)amines, with methoxy groups in different positions on the benzene ring (p-OMe, m-OMe, or o-OMe), can significantly alter the physical and chemical properties of spiro-OMeTAD. Jeon et al. investigated how these derivatives of spiro-OMeTAD affect the performance of PSCs [46]. m-OMe tended to withdraw electrons, while p- and o-OMe showed electron-donating behavior; therefore, the PSCs using m-OMe spiro-OMeTAD showed low performance [46]. The investigation showed that p- and o-OMe at a ratio of 2:2 in spiro-OMeTAD showed a significant improvement in PSCs with a PCE of ~16.7% and FF of 77.6%, while only p-OMe spiro-OMeTAD exhibited a PCE of 15% and FF of 71.1%. Other spiro-OMeTAD derivatives could formed by replacing the spirobifluorene core with cores of pyrene, thiophene, tetraphenylethene, etc., which would provide a different energy configuration for mobility, HOMO, and LUMO levels [47,48,49,50,51,52]. Saliba et al. (2016) utilized a fluorene–dithiophene (FDT) core substituted with donor groups to create a new hole transport material (HTM) with a high adiabatic oxidation potential of approximately 5.15 eV, surpassing that of spiro-OMeTAD at approximately 4.98 eV. This higher potential indicated increased stability. FDT-based HTM in perovskite solar cells (PSCs) exhibited improved $J_{SC}$ and $V_{OC}$ when compared to spiro-OMeTAD-based devices, resulting in an impressive PCE of approximately 20.2% and enhanced long-term stability [53]. Zhang et al. (2018) employed spiro[fluorene-9,9′-xanthene] (SFX) as a new core for synthesizing new HTMs, X26 and X36, which exhibited higher conductivity than spiro-OMeTAD by 2–5 times [54]. The X26-based devices showed a high PCE of ~20.2%. Moreover, under controlled humidity of ~20%, the devices could maintain a PCE of 18.8% after 5 months, indicating the highly stability of the new HTMs. Jeong et al. (2022) successfully fabricated spiro-Naph as an HTM for PSCs, which exhibited an impressive PCE of 24.43% [55]. Moreover, the devices showed excellent stability and thermal endurance at an elevated temperature of 60 °C after 400 h. The improvement came from a significantly high hole mobility of ~8 × 10^3^ cm^2^ V^−1^ s^−1^ and the HOMO level of spiro-Naph was closer to the HOMO of perovskite materials; therefore, the transportation of holes was facilitated. In general, the use of different cores leads to the tuning of energy level of the HTM, offering more flexibility in achieving a low energy barrier for efficient hole transport within the solar cell configuration [56]. In addition, improvement in hole mobility is also enhanced with the introduction of more benzene rings with functional groups.

### 3.2. PTAA

Poly[bis(4-phenyl)(2,4,6-trimethylphenyl)amine] (PTAA) has attracted interest as a potential HTM in a variety of optoelectronic devices, including solar cells. PTAA is a type of polymer that is often used as a replacement for other hole transport materials, such as spiro-OMeTAD, due to its favorable electronic properties and ease of processing [57]. PTAA has excellent solubility in a variety of organic solvents and an amorphous, thermally stable shape. Neither the melting phase nor the glass transition were visible in pure PTAA up to 300 °C [58]. When LiTFSI and tBP were used to dope all HTLs, PTAA outperformed a variety of other semiconducting polymers, including spiro-OMeTAD, to achieve an efficiency of 12% in PSCs in 2013 [59,60]. Similar to spiro-OMeTAD, PTAA only functions well in the presence of supplements and causes instability problems [10]. The performance of PSCs with pristine PTAA could reach a PCE of 18.11% [61], while the combination of PTAA and dopants such as Li-TFSI could lead to a PSC with a PCE of 19.7% for 1 cm^2^ cells [62]. The optimal doping concentrations for PTAA-based PSCs are, however, roughly four times less than the typical concentrations of tBP and LiTFSI added to spiro-OMeTAD [63]. PTAA can be synthesized using simpler and more scalable methods compared to spiro-OMeTAD, potentially leading to lower production costs. Other hole-conducting polymers did not interact with the perovskite at the interface as strongly as PTAA, which may have improved hole transport [18]. PTAA is considered to be more environmentally friendly than spiro-OMeTAD, which contains heavy metal elements. Although PTAA is still expensive (19.80 USD/mL), its cost is still lower than that of spiro-OMeTAD [33].

**Figure 4:**
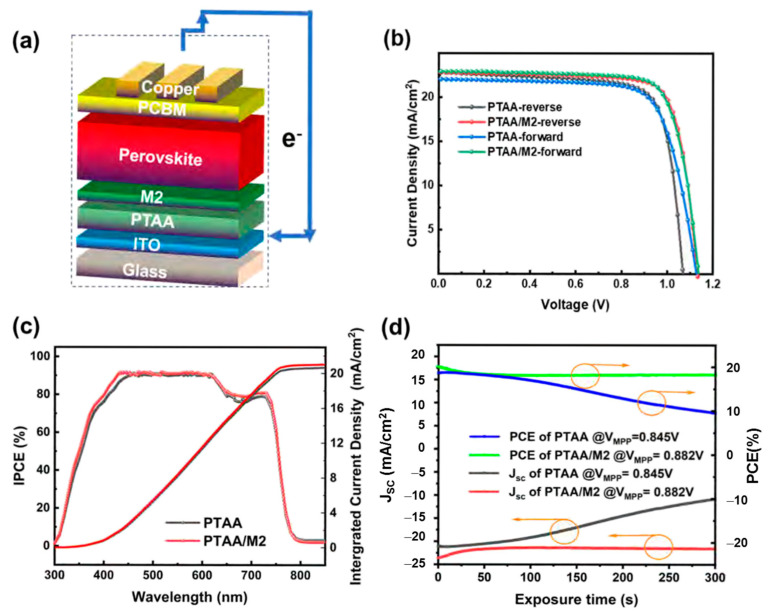
(**a**) The structure of PSCs based on PTAA/M2, (**b**) current density–voltage characteristics, (**c**) incident IPCE spectra, (**d**) stabilized power output, tracked at MPP under AM 1.5G illumination. Reprinted with permission from ref. [57] Copyright 2022, American Chemical Society.

While PTAA has shown promise, its hole mobility and charge extraction efficiency can still be lower than those of spiro-OMeTAD in some cases [64]. Greater quality and defect passivation in the perovskite at the interface was partly responsible for PTAA’s lower rates of recombination compared to other widely used HTMs [65]. Similar to spiro-OMeTAD, despite the strong thermal durability of pure PTAA, thermal strain does cause perovskite cracks in doped PTAA-based PSCs, leading to a 60% efficiency reduction [66]. PTAA is also sensitive to moisture and is not a reliable barrier to ionic diffusion [18]. Another limitation of PTAA comes from its hydrophobicity, which prevents PTAA film from developing high-quality perovskite [57,67]. Bagheri et al. (2020) overcame this limitation when applying PTAA in the inverted PSC (p-i-n) architecture by preparing the PTAA layer with a quick UV treatment before perovskite was deposited [67]. The PTAA layer’s PCE was 19.17% for a 0.09 cm^2^ active area due to improvements in the optical characteristics, grain size, and the decrease in recombination centers. The device also kept more than 75% of its initial efficiency after 1400 h of storage under atmospheric conditions with an average relative humidity (RH) of 50% [67]. In other study, Li et al. (2022) modified the surface of PTAA with 4,4′,4″-(1-hexyl-1H-dithieno[3′,2′:3,4:2″,3″:5,6]benzo[1,2-d]imidazole-2,5,8-tryl)tris(N,N-bis(4-methoxyphenyl)aniline) (denoted as M2) [57]. Due to improved PTAA hydrophobicity, M2’s presence promoted the development of perovskite film. PTAA/M2 also had greater hole conductivity and mobility compared to pristine PTAA. The application of M2 increased the PCE of inverted PSCs from 18.67% to 20.23% (Figure 4) [57]. Spiro-OMeTAD is commercially available and widely used in research and industry. PTAA might have limited commercial availability. Spiro-OMeTAD has been extensively used as a benchmark material, and its performance has been well characterized. PTAA is still being evaluated, and its performance might not be as standardized. 

### 3.3. PEDOT:PSS

PEDOT:PSS offers high charge carrier mobility, an adequate band gap, optimal spectrum alignment, and an inexpensive price [14]. Additionally, PEDOT:PSS offers excellent formability and strong transparency in the visible region [68]. PEDOT:PSS has been employed as an HTM in OSCs, facilitating the efficient transport of positive charge carriers (holes) from the photoactive layer to the electrode. While primarily used as a transparent conductive electrode in PSCs, PEDOT:PSS has also been explored as an HTL in certain device architectures. It can effectively transport holes from the perovskite absorber layer to the electrode, contributing to enhanced charge extraction and overall device performance.

However, PEDOT:PSS still has some limitations. PEDOT:PSS contains a polystyrenesulfonate component that is acidic, which can cause the degradation of adjacent layers and impact device stability. Low $V_{OC}$ (<1 V) is produced as an outcome of the energy gap between PEDOT:PSS (−5.12 eV) and CH_3_NH_3_PbI_3_ (−5.4 eV) perovskites [68]. Achieving optimal energy level alignment between PEDOT:PSS and the active layer can be challenging, affecting charge extraction and recombination dynamics. The existence of the weak ionic conductor PSS limits and impairs charge transfer in PEDOT:PSS [69]. The PSS in PEDOT:PSS can be removed using a simple solvent engineering method (ethylene glycol and methanol). This strategy can increase the PCE of PSC devices to 18.18% [69]. While PEDOT:PSS is an effective conductor, its interfacial properties can impact electron blocking and recombination mechanisms [70]. Inside the PEDOT:PSS layer, poor hole migration can originate from imperfections in the PEDOT:PSS film’s microstructure and the gradient of electrical conductivity between the surface and the bulk. This causes an imbalance in carrier charge transfer and the buildup of charge carriers, which finally leads to low FF and large leakage of current [68]. To increase the Fermi level of PEDOT:PSS by up to 500 meV, Chin et al. (2022) dedoped it using NaOH. Enhanced photoluminescence duration and greater photovoltage of the surface, which resulted in higher $V_{OC}$, fill factor, and PCE, were signs that recombination losses were significantly reduced at the dedoped PEDOT:PSS/perovskite interface [70]. PEDOT:PSS is hygroscopic, meaning it can absorb moisture from the environment, which might affect device performance and stability. The moisture sensitivity of PEDOT:PSS can be overcome by the introduction of a crosslinking agent combined with surface treatment [71]. The PEDOT:PSS’s crosslinking system converted its naturally water-soluble features into a water-resistance characteristic, preventing water penetration. Additionally, MeOH treatment enhanced PEDOT:PSS’s conductivity and decreased its surface roughness by eliminating surface traces [71].

### 3.4. P3HT

Due to its stability, low cost, high hole mobility (0.1 cm^2^ V^−1^ s^−1^), and high efficiency, the conjugated polymer poly(3-hexylthiophene) (P3HT) has received extensive study and is utilized as an HTM in organic solar cells (OSCs) [72]. P3HT has a conjugated polymer backbone, which makes it possible for long-range electron delocalization to occur. This results in good charge carrier mobility and efficient charge transport. P3HT can be processed from solution, enabling its incorporation into scalable and cost-effective manufacturing methods, such as spin-coating and printing. P3HT exhibits reasonable electrical conductivity, making it suitable for charge transport within the active layer. Due to its resistance to temperatures in the range of −80 °C to 100 °C, low oxygen penetration, and extreme hydrophobicity, P3HT is more stable than spiro-OMeTAD [73]. Application of P3HT in mesoporous PSCs on solid substrates produced a PCE of 22.7% when a layer of wide-bandgap halide perovskite was employed on top of a layer of narrow-bandgap perovskite [33].

While P3HT has relatively good hole mobility compared to some other organic materials, it still falls short of the hole mobility exhibited by some inorganic materials. P3HT-based solar cells can experience energy losses due to charge recombination and the formation of non-radiative recombination pathways. Low PCE was caused by poor interaction and significant recombination at the junction between perovskite and P3HT [72]. P3HT has a relatively low absorption coefficient, which can limit its ability to efficiently absorb photons and contributes to photocurrent generation. P3HT-based solar cells can face challenges related to long-term stability under environmental conditions and operational stress. P3HT-based organic solar cells have demonstrated moderate efficiency levels compared to some other organic and inorganic solar cell technologies. A PCE as low as 16% may be attained with pure P3HT as an HTM [74,75]. Different additives like BTCIC-4Cl, copper(I) thiocyanate (CuSCN), n-hexyl trimethyl ammonium bromide (HTAB), gallium (III) acetylacetonate (Ga(acac)_3_), and SMe-TATPyr were used to fix problems at the interface between P3HT and perovskite [76,77]. Recently, Xu et al. (2022) used 2-((7-(4-(bis(4-methoxyphenyl)amino)phenyl)-10-(-2-(2-ethoxyethoxy)ethyl)-10H-phenoxazin-3-yl)methylene)malononitrile (MDM) as a molecular link for stable and effective PSCs (PCE of 22.87%, $V_{OC}$ of 1.15 ± 0.02, and fill factor (FF) of 75.02 ± 3.09%). The triphenylamine group of MDM could create π–π stacking with P3HT, creating a charge transfer pathway, whereas the malononitrile group of MDM could bond the perovskite surface. Additionally, MDN greatly reduced recombination and passivated flaws. A total of 92% of the initial PCE of the MDN-P3HT-based PSC device was preserved even after aging for two months at a RH of 75%. Additionally, the PCE remained constant after 500 h of operation under a single sun illumination at the maximum power point (MPP, −45 °C in N_2_) [72].

### 3.5. Other OHTMs

In addition to the OHTMs mentioned, many novel dopant-free OHTMs have also been applied as HTLs for LCSCs. For instance, poly(2,7-carbazole) (PCz) has been used as an HTL. PCz, an aromatic heterocyclic conducting polymer that contains nitrogen, has fast charge mobility, outstanding morphological stability, and great optoelectronic characteristics [78]. Its use in PSCs results in a device PCE of 18.04%. Moreover, it has been demonstrated that PCz functions as a strong barrier and adequately protects the perovskite surface, resulting in highly stable PSC devices [79]. Recently, Wang et al. (2022) drove the self-assembling of carbazole through hydrogen bonding. In comparison to hydrogen bonding-free devices, n-i-p PSC devices based on the hydro-functional HTM showed improved hole extraction reaction, inhibited interfacial charge recombination, decreased hysteresis effect, and significant raises in $V_{OC}$, FF, and overall PCE [80]. Organic compounds that are heterocyclic and have rings with carbon and sulfur atoms, like benzothiophene, 1,3-bis(4-(2-ethylhexyl)-thiophen-2-yl)-5,7-bis(2-ethylhexyl)benzo[1,2-c:4,5-c’]-dithiophene-4,8-dione (BDD), dithieno[3’,2’:3,4:2″,3″:5,6] benzo[1,3-c][1,2,5]thidiazole (BTT), random copolymer (RCP) of benzo[1,2-b:4,5:b’]dithiophene (BDT), and 2,1,3-benzothidiazole (BT), have also been applied as HTLs [19,81,82,83]. He et al. (2023) suggested a solvothermal treatment for benzothiophene to obtain benzothiophene carbon dots (CDs) [81]. The new HOMO value of the obtained material more closely resembled the perovskite valence band. Consequently, these CD-modified PSCs exhibited an acceptable power conversion efficiency (13.22%). The presence of benzothiophene CDs also improved the crystallinity and hydrophobicity of the perovskite film. The increase in hydrophobicity could be related to the generation of a Pb–S bond at the interface between perovskite and benzothiophene CDs [81]. Recently, a PCE of 20.9%, $V_{OC}$ of 1.14 V, $J_{SC}$ of 23.4 mA cm^−2^, and FF of 78.9% were achieved by n-i-p PSCs based on BTT, while these values for devices based on BDD were 16.3%, 1.12 V, 23.2 mA cm^−2^, and 62.8%, respectively [19]. A similar HOMO level of −4.9 eV and high hole mobility of over 5 × 10^−4^ cm^2^ V^−1^ s^−1^ were observed for BDD and BTT [19]. Meanwhile, PSCs with RCPs of BDT and BT as HTMs could reach a PCE of 17.3%. The high mobility and deep HOMO energy level were responsible for the observed PCE. A hydrophobic polymer layer and the avoidance of hygroscopic or deliquescent dopants also increased the long-term PCE. At 75% humidity, PSCs with random copolymers continued to function at their initial PCE for more than 1400 h, but HTM devices with additives had a reduced PCE after 900 h [82]. The remarkable studies that have utilized organic materials as HTMs are summarized in Table 1.

There are some strategies to improve the performance of LCSCs with organic materials as HTLs. Dopant-free organic materials should be considered because they can avoid the use of additives that can increase the instability and toxicity of OHTLs. Some materials have been introduced in the literature, such as D-A-π-A-D-type DTP-C6Th, 1,10-phenanthroline (YZ22), DTB-FL, Ni phthalocyanine (NiPc), 2DP-TDB, phenanthrocarbazole 6 (PC6), and Mesh TABT [84,85,86,87,88]. However, even on a lab scale, none of them can actually take the place of doped spiro-OMeTAD [72]. In addition, inorganic materials such as carbon materials (such as carbon nanotubes or graphene), metals (such as aluminum and copper), metal oxides (such as NiO_x_ and MoO_x_), or metal compounds (such as CuSCN and CuPc) can be applied to combine the advantages of both inorganic and organic materials [89,90,91,92,93]. PSC efficiency and stability have recently been improved by combining tBP- or Li-TFSI-doped spiro-OMeTAD with fluorinated graphene (FG). The PCE of the PSCs reached 21.92% and further increased to 23.14% when a 2D interfacial layer was installed. FG increased the hydrophobicity of the HTL and enhanced lithium ion reduction in the perovskite layer, in addition to increasing the hole mobility of spiro-OMeTAD. During a 2400 h test in ambient circumstances with 25% RH, the FG-incorporated cell exhibited higher stability, keeping 90% of its initial PCE [93]. However, the introduction of inorganic materials could lead to several negatives, such as high price, low transparency, and possible corrosion [94]. In addition, most inorganic materials are difficult to deposit on top of perovskite [18]. There are also some unique strategies to improve the performance of PSCs using OHTMs as HTLs. For example, Zhang et al. (2023) intertwined single-walled carbon nanotube (SWCNT) electrodes with dopant-free PTAA and applied them in dual roles as the HTL and electrode in PSCs [95]. The PSCs with these dual-role electrodes achieved a better PCE and stability compared to PSCs using LiTFSI-doped spiro-OMeTAD [95]. 

## 4. Inorganic Hole Transport Layer (IHTL)

### 4.1. Graphene Oxide and Carbon Derivatives

**Figure 5:**
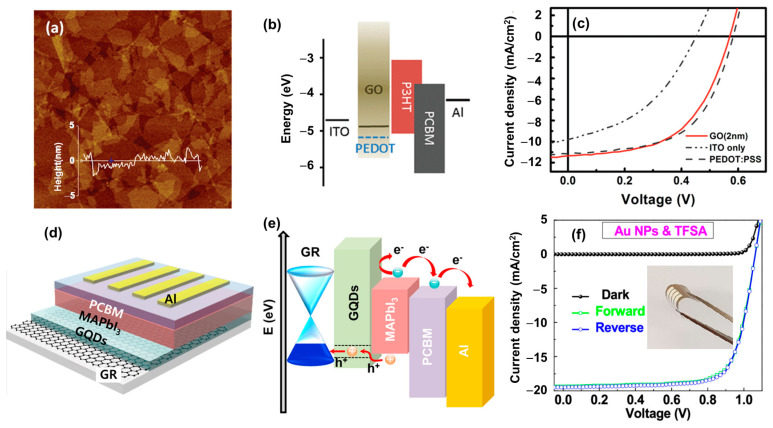
(**a**) Atomic force microscopy image of GO; (**b**) energy band alignment of GO in OSC structure; (**c**) J–V curves of GO-, PEDOT:PSS-, and bare ITO-based OSCs. Reproduced with permission from ref. [101] Copyright 2010, American Chemical Society. (**d**) Structure; (**e**) energy band alignment; and (**f**) J–V curves of flexible GQDs and graphene-based MAPbI_3_ PSCs. Reprinted with permission from ref. [114] Copyright 2019, American Chemical Society.

Graphene oxide (GO) and reduced GO (rGO) have unique optical and electrical properties such as high transparency (94%), high conductivity (0.45 S/cm), easy processing, and an atomic-scale thin layer [96]. GOs have the structure of graphene and contain mixed sp2 and sp3 hybridizations of carbon and carbon–oxygen (C–O, C=O, C–OH) covalent bonds, which not only provide a stable structure but also allow tunability of the band gap and work function [97,98]. As reported in the literature, the work functions of GO and rGO are around 4.0–5.0 eV depending on the type of dopant, which is adjustable to fit with the requirements of HTLs in OSCs [99,100,101]. Li et al. (2010) proposed a solution process to use GO as an HTL in OSCs with P3HT/PCBM as the active material [101]. Using the spin-coating method, very thin layers were obtained with various thicknesses ranging from 2 to 10 nm and a smooth surface with a low roughness of under 1.4 nm, as illustrated in Figure 5a. The method indicated easy processing for a high-quality, thin GO-based HTL. Figure 5b shows the well-matched band gap alignment of GO in the structure of OSCs. Moreover, GO-based HTLs in OSCs showed comparable performance with traditional cells, which promises to be a potential HTM, as shown in Figure 5c. However, GO was pointed out to have low ohmic contact with other layers, which reduces the efficiency of the hole transport process. Thus, GO was reduced to rGO or mixed/doped with other materials to overcome this drawback [102]. Yu et al. (2014) demonstrated the use of GO with PEDOT:PSS composite, which could enhance the properties of OSCs, and achieved a high PCE of 8.21% in comparison with 7.04% for bare PEDOT:PSS [103]. Meanwhile, Liu et al. (2012) sulfated the GO surface (S-GO) by fuming with sulfuric acid and employed S-GO as an HTL to replace PEDOT:PSS. The S-GO-based devices had a comparable PCE to PEDOT:PSS of ~4.4% [104]. Stratakis et al. (2014) used a laser method to form chlorine-doped GO with a work function of 5.23 eV as an HTL in OSCs, significantly outperforming the reference PEDOT:PSS [105]. Jeon et al. (2014) used a spraying method with a heating process to form a coated GO film on the substrate, resulting in moderately thermally treated rGO as the HTL [106]. Metal/halogen, N dopant, or composite routes for GO/rGO were also investigated to find the best combination of this HTL in OSCs and PSCs, such as Cu, Cl, F, and polyaniline [107,108,109,110,111]. rGO was also used as an HTL in PSCs, which even showed better performance compared to PEDOT:PSS, where the PCE reached ~10.8% [112]. Lou et al. (2017) employed a GO-modified PEDOT:PSS surface, which improved the wettability of the HTL layer; therefore, the PSC performance was improved with a PCE of 15.3% [113]. 

In addition, carbon derivatives with a graphene-core structure was also promising to exhibit similar behavior to GO/rGO [114,115,116]. Graphene quantum dots (GQDs) or carbon dots are well known in optical applications, owing to their fluorescence and electrical properties, which are widely used as biomarkers and light emission materials [117,118,119]. GQDs are easily fabricated via hydrothermal, microwave-assisted, or solvothermal methods [119,120,121]. Li et al. (2013) used a one-step acid treatment method to synthesize GQDs from carbon fibers, which were then employed as HTLs in OSCs [122]. The GQD-based devices showed a significant improvement in producibility and lifetime stability, while the PCE was comparable to that of PEDOT:PSS-based devices. Shin et al. (2019) reported employing GQD HTLs in PSCs with Au nanoparticles (NPs), which achieved a PCE of ~15.5% and excellent bending stability of flexible PSCs (retaining 70% PCE after 3000 bending cycles), as shown in Figure 5d,e [114]. Ali et al. (2023) fabricated vertically aligned CNT thin film as an HTL to protect against direct contact of PEDOT:PSS with the current collector [123]. The CNTs in composition with PEDOT:PSS could improve the stability and increase the conductivity of the HTL. Owing to their high physicochemical properties, low dimensional GO/rGO and carbon derivatives could provide a high mobility, smooth surface with a roughness of a few nm and a huge improvement in stability. However, the low wettability limits their application; therefore, surface modification and functionalization are useful for resolving these issues.

### 4.2. Metal Oxides

**Figure 6:**
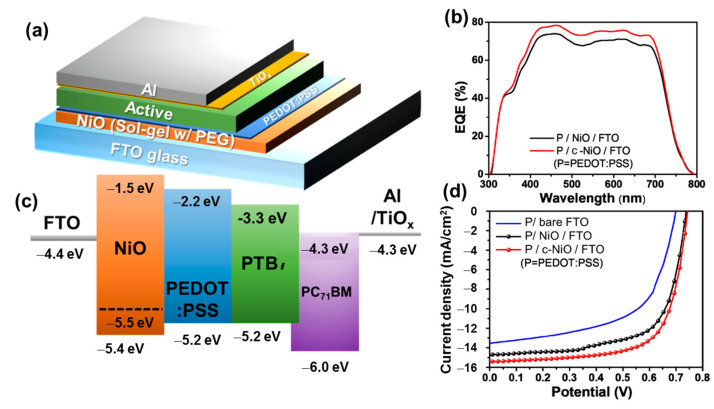
(**a**) Cell structure; (**b**) external quantum efficiency; (**c**) band alignment of energy; and (**d**) J–V curves of NiO/PEDOT:PSS HTL-based OSCs. Reproduced with permission from ref. [136] Copyright 2019, MDPI.

Metal oxide (MO) materials are promising to overcome the challenges of organic HTMs due to their inherent physical/chemical stability and charge conductivity [124]. Especially, transition metal oxide materials have special properties due to their electron structure, which is formed by partially filled in d orbitals and the outer shell of valence electrons [125,126]. Nickel oxide (NiO_x_) is a p-type direct band gap semiconductor (Eg ~ 2.7 eV) [127]. The valance band of NiO_x_ is well aligned with the HOMO level of many conjugated polymers, and the conduction band is high over the LUMO of the active material [128,129]. Therefore, the band structure of NiO_x_ matches not only the HTL but also the electron-blocking layer (EBL). Due to its high stability, NiO_x_ can be prepared by a variety of methods, such as sol-gel, electrodeposition, oxidation, and spray [128,130,131,132,133,134]. Parthiban et al. (2017) produced NiO_x_ by a solution process, with a valance band of 5.3 eV, which could improve the stability of OSCs and exhibited a comparable PCE to PEDOT:PSS-based devices [135]. Kim fabricated a compact NiO film on an FTO electrode, which could improve the PCE from 5.68% to 6.91%, and the use of both NiO and PEDOT:PSS could boost the PCE to 7.93%, as shown in Figure 6a–d [136]. In addition, NiO_x_ is commonly used as an HTL in PSCs due to its own properties, low electrode polarization, and suitable surface for growth of the perovskite layer [137]. Liu et al. (2018) employed NiO_x_ as an HTL in methylammonium (MA)-mixed formamidinium (FA) halide (I, Br, Cl) perovskite solar cells, which increased the PCE to 20% [138]. The role of NiO_x_ is not only to be a simple HTL layer but also to enhance the open voltage of PSCs [139]. NiO_x_ has been modified by various techniques, such as metal doping (Cu, Li, Mg, Cs, and Zn), surface modification, and the UV-ozone technique, to obtain suitable band alignment with OSC structures [140,141,142,143,144,145,146]. 

Molybdenum oxide (MoO_x_) and tungsten oxide (WO_x_) are also commonly used in OSCs. MoO_x_ and WO_x_ are n-type semiconductors with a wide band gap of ~3.0 eV, a suitable work function of ~5.0 eV, and high surface energy, which would be a potential HTL in OSCs and PSCs [147,148]. A wide band gap allows light to easily pass through. Also, it is easy to make thin films of MoO_x_ and WO_x_ using physical or chemical methods. This means that MoO_x_ and WO_x_ can be used in both normal and inverted structures of OSCs and PSCs. Jasieniak et al. (2012) proposed a solution process to create MoOx thin film using the electrospray ionization method as an HTL in OSCs [149]. The presence of Mo(V) in MoO_x_ revealed that increasing the ratio of Mo^5+^/Mo^6+^ (from 0.02 to 0.25%) by annealing treatment could improve the performance of OSCs from low to comparable with PEDOT:PSS-based cells. Yang et al. (2018) prepared an n-doped MoO_x_ thin film with a conductivity as high as up to 11 S/cm by introducing a part of Mo(V) to Mo(IV) [150]. The OSC devices with a 10 nm thin film of n-doped MoO_x_ as an HTL showed a high PCE of 11.4%. Modification by the dopant effect introduced a work function of ~4.9 eV, which reduced the energy barrier for the extraction of holes from the active material to the current collector and enhanced the performance and stability of OSCs. Liu et al. (2023) reported that the use of MoO_x_ as an HTL could boost the PCE of OSCs to 16.8% and provided excellent air stability over 600 h compared to the PCE of ~16.4% and the lifetime of 70 h of PEDOT:PSS-based devices at 85 °C [147]. Han et al. (2009) employed WO_3_ thin film as an HTL, which enhanced the performance of OSCs [151]. The WO_3_ amorphous film could reduce the roughness of the thin film to ~0.88 nm. Further, the low surface energy of WO_3_ led to the uniform growth of P3HT; therefore, the recombination of charge carriers was reduced. In a similar approach, amorphous WO_x_ was widely used as an HTL in OSC or PSC devices [152,153,154]. Stubhan reported a low-temperature process to fabricate WO_3_ as an HTL without various treatments, such as oxygen-plasma or annealing treatment. Moreover, Liu et al. (2018) also used the combination of WO_3_ with PEDOT:PSS to reduce the hole transport barrier to the ITO collector [155]. Owing to the high band gap and tunable work function via the dopant or oxygen vacancy, WO_x_ could also be used as an ETL in PCSs or OSCs [156,157]. 

Besides NiO_x_, WO_x_, and MoO_x_, metal oxides of Cu, Cr, Co, Ti, and Ir were also used as HTLs, such as CuO_x_, Cu_2_O, and Co_3_O_4_, or binary metal oxides such as NiCo_2_O_4_, CuCrO_2_, ZnCo_2_O_4_, and TiO_2_-IrO_x_ [158,159,160,161,162,163,164,165,166,167,168]. Yu et al. (2016) demonstrated a solution process of CuO_x_ film for OSCs [164]. The work function of CuO_x_ was tunable via oxidation treatment with H_2_O_2_ or UV-ozone treatment, which increased from 5.06 to 5.45 eV. The CuO_x_ could boost the PCE of OSCs up to 8.68% (10% higher than PEDOT:PSS-based devices). Zhang et al. (2019) used the Mg metal as a dopant in CuCrO_2_ nanoparticles as an HTL in OSCs and PSCs [169]. The presence of Mg not only contributed to decreasing the size of CuCrO_2_, but also tuned the work function of this material; therefore, the performance of OSCs and PSCs was improved, especially the stability of PSCs (lifetime over 80 h). Liu et al. (2019) used Cu_2_O quantum dots by the simple modification method, which could be placed on top of the perovskite layer for inverted PSCs [158]. In general, perovskite material is quite sensitive and can be destroyed by moisture or ionic factors. In this case, Cu_2_O quantum dots were not only compact to the perovskite layer but also protected it, achieving a high PCE of 18.9%. Papadas et al. (2018) demonstrated the use of spinel NiCo_2_O_4_ HTM in PSCs [162]. Metal oxides are highly stable materials, benefiting the long-term stability of materials. However, they have drawbacks such as high processing temperatures and low electronic mobility [170]. Therefore, a low processing technique combined with a doping material to increase the electronic mobility is required to employ metal oxides as HTLs.

### 4.3. Transition Metal Sulfides

**Figure 7:**
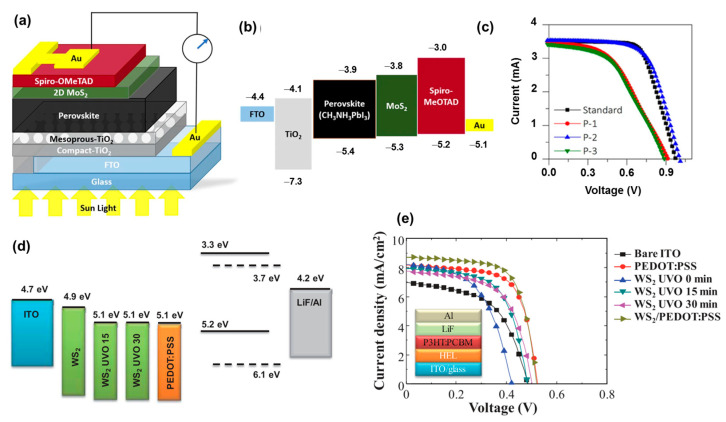
(**a**) Scheme of PSCs based on 2D MoS_2_; (**b**) energy band alignment of different layers in PSC; (**c**) J–V curves of devices with different treatments P1, P2, and P3, corresponding with MoS_2_ on perovskite without annealing, annealing MoS_2_ on pre-annealed perovskite, and annealing MoS_2_ on perovskite without annealing, respectively. Reproduced with permission from ref. [185] Copyright 2020, Springers Nature. (**d**) Energy band alignment work function of WS_2_ with/without UV treatment and PEDOT:PSS in OSCs; and (**e**) J–V curves of devices based on WS_2_-UVO and PEDOT:PSS as HTLs. Reprinted with permission from ref. [188] Copyright 2014, Wiley-CH.

Transition metal oxides (TMOs) and transition metal sulfides (TMSs) are also attractive to researchers due to their intrinsic properties. Owing to the higher electron affinity of the S atom compared to the O atom, most TMSs have higher conductivity than TMOs, and the band gap of TMSs is also complex and has a variety of energy band structures [171,172]. Among TMSs, layered structures such as MoS_2_, WS_2_, TiS_2_, and TaS_2_ (transition metal dichalcogenides, TMDs) are widely applied in optical/electrical devices due to their superior properties [173,174,175]. TMDs have a layered structure, with each layer consisting of a transition metal layer (Mo, W, Ti, and Ta) sandwiched between chalcogenide layers (S) and stacked by weak Van der Waals forces to form the bulk structure [176,177]. Therefore, they can easily be fabricated by physical or chemical methods [178,179]. Especially, the energy band structure strongly depends on the number of layers. In the bulk structure, TMDs have a low indirect band gap of ~1.2 eV. When they become single layers, the band gap can be opened to ~2.0 eV as a direct band gap, which not only tunes the band structure but also improves the optical/electrical properties. Moreover, the different stacking of layers leads to different phases, where the 2H phase (hexagonal structure) is semiconductive and the 1T phase (trigonal structure) is metallic [180,181,182,183]. Due to the low band gap, TMDs could partially hinder light from passing through; therefore, the use of a very thin layer (increasing transparency) is important [184]. Liang et al. demonstrated MoS_2_ nanoflakes as an HTL buffer layer of PSCs (glass/FTO/compact-TiO_2_/mesoporous-TiO_2_/FA_85_MA_15_PbI_85_Br_15_/2D MoS_2_/Spiro-OMeTAD/Au), which exhibited better stability under continuously illumination, as shown in Figure 7a–c [185]. Wang et al. (2018) combined MoS_2_ with PEDOT:PSS as a hybrid HTL for PSCs [186]. The MoS_2_ nanoflakes improved contact between the composite HTL and the ITO electrode, enhanced the stability of devices up to 28 days, and retained 95% of the initial PCE (18.5%). The pure MoS_2_ and WS_2_ had work functions that were quite high at ~4.6 eV; therefore, intrinsic TMDs may not effectively transport charge carriers [187,188,189,190]. Le et al. (2014) used UV-ozone treatment to partially oxidize MoS_2_/WS_2_ to MoS_2•_MoO_x_ and WS_2•_WO_x_, reducing the work function to ~5.1 eV, which was compatible with the HOMO level of P3HT:PCBM materials, as shown in Figure 7d,e [188]. The UVO-treated TMDs as HTL devices showed a comparable PCE with PEDOT:PSS-based devices. Xing et al. reported the use of MoS_2_ quantum dots with UV-ozone treatment as an HTL in OSCs [191]. The quantum dots provided a low roughness of ~1–2 nm, which benefited the coating process of other layers and reduced the resistance of the contact surface. Additionally, the UV-ozone-treated MoS_2_ quantum dots had a work function of ~4.9 eV, which was more suitable to transporting holes from P3HT:PCBM. Adilbekova et al. (2020) employed MoS_2_ and WS_2_ nanosheets as HTLs in OSCs, which showed a similar PCE to devices based on PEDOT:PSS [192]. The UV-ozone treatment was also performed on WS_2_, TiS_2_, and TaS_2_ materials, especially TaS_2_, which had high conducting properties and was also employed as an ETL layer [187,188,189]. MoS_x_ and WS_x_ nanodots, or the amorphous structure of MoS_x•_MoO_x_ and WS_x•_WO_x_, were also used to make the smooth surface of an HTL by simple solvothermal synthesis [193,194]. This behavior was derived from the partial oxidation of the TMD surface, which not only induced the charge carrier from binding with oxygen but also formed a thin layer of TMD, benefiting the conductivity and stability of the HTL.

TMSs that are not layered materials, such as NiS or CuS, also possess unique properties. CuS and NiS are both p-type semiconductors with work functions ranging from 4.9 to 5.1 eV, which are promising for transporting holes to current collectors [195]. Rao et al. (2016) demonstrated a simple solution process to obtain CuS NPs, which could be a highly stable HTL in PSCs [195]. The CuS thin layer was uniformly coated on the ITO/glass substrate and did not affect the transparency of this substrate. Moreover, the CuS surface was also compact for growth of the active material, lessening the barrier to carrier injection at the interface and improving the performance of PSCs. Li et al. (2020) described the procedure for a room-temperature solution to make p-type CuS_x_ thin film as a stable HTL [196]. Tirado et al. (2019) revealed that p-type CuS had a metallic character in the valence band under light irradiation, benefiting the transport of holes and effectively blocking electrons. Thus, it could be used to replace an expensive ETL layer such as spiro-OMeTAD [197]. CuS could also be combined with GO or modified in various compositions, such as CuSCN, Cu_2_CdSnS_4_, and Cu_2_SnS_3_, to improve the hole transport characteristics, surface engineering, and compactness of other kinds of active materials [198,199,200,201]. While CuS has a low band gap of ~1.5 eV, NiS possesses a wide range of band gaps from 1.9 to 2.4 eV, depending on the synthesis conditions and capping agents [202,203]. Recently, Hilal et al. (2019) reported the use of NiS nanoflowers as an HTL for OSCs [204]. NiS nanoflowers possess a work function of 5.04 eV, which is reasonable for an HTL layer. Pitchaiya et al. (2018) used NiS-carbon composites as an HTL in PSCs and showed a PCE of 5.2% [205]. However, the transparency of NiS (~70%) is still an obstacle to this HTL [204]. The benefits of metal sulfide materials are their high charge carrier properties, and the S atom is compactable with both inorganic and organic active materials; therefore, they are widely applied in OSCs and PSCs. Owing to their high electronic conductivity and low band gap energy, TMDs are used in inverted structures. However, adjusting the material thickness and UV-ozone treatment could modify their surface and work function to match designated structures in conventional cells. The 1T phase of TMDs is a promising HTL; however, it can gradually transfer back to the 2H phase while suffering a high working temperature. Non-layered TMSs are more stable and can be used as HTLs; however, due to their low transparency, they are generally used as a doping material or thin support layer with another HTL [206].

### 4.4. Organometallic Materials

**Figure 8:**
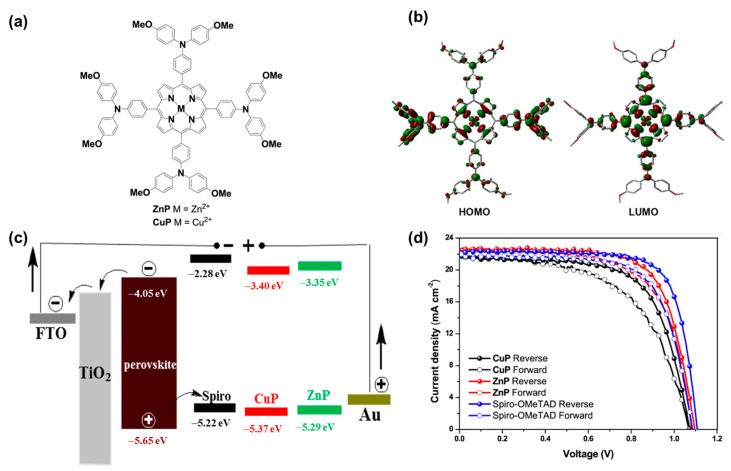
(**a**) Chemical structures of ZnP and CuP with (**b**) their frontier orbitals; (**c**) energy band alignment of perovskite and HTLs; and (**d**) J–V curves measured in reverse and forward voltage scans of CuP, ZnP, and spiro-OMeTAD-based HTL PSC devices. Reproduced with permission from ref. [213] Copyright 2017, American Chemical Society.

**Figure 9:**
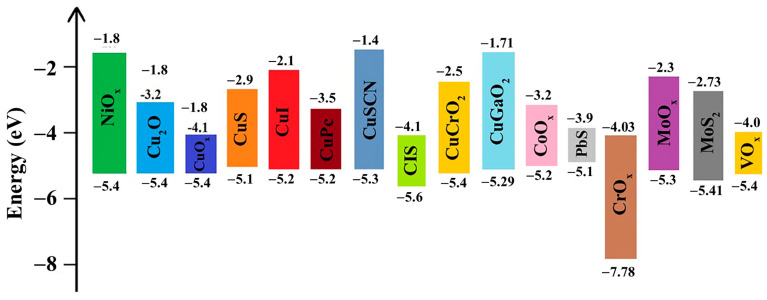
Energy level diagrams for commonly used inorganic HTLs in PSCs. Reproduced with permission from ref. [207] Copyright 2021, Wiley-CH.

Recent research on organometallic materials (OMs) has shown the interestingness of organic and metallic properties in a composition. OMs are believed to solve the drawbacks of organic materials, such as low-charge carriers and poor stability, and the drawbacks of inorganic materials, achieving compatibility through surface engineering [34,94,207]. Recently, metal phthalocyanines (MPcs) have received significant attention as OMs for HTLs of PSCs [208]. MPcs have a novel structure with a centered metal atom, which can be easily obtained by a simple reaction of a phthanonitrile-based material with metal salts such as a metal chloride or acetate. MPcs have unique properties, such as a low band gap, high physical/chemical stability, and high carrier mobility [208,209,210,211]. Guo et al. (2017) synthesized ZnPc and CuPc and proposed their use as HTMs in PSCs [208]. ZnPc-based devices achieved high stability after over one month with a comparable open voltage of ~1.071 V to Spiro-OMeTAD at 1.107 V. The PCE of devices based on MPc was ~14.4%, which was still lower than that of Spiro-OMeTAD-based devices (18.9%), but significant improvements in stability and cost-effectiveness would lead this material to becoming a potential HTL, which is also an EBL in PSCs. Using a similar approach, Ni, Zn, and Co metals were also synthesized as MPc materials. Cheng et al. (2017) proposed the use of NiPc and V_2_O_5_ as a comparative HTL to spiro-OMeTAD, achieving a PCE of up to 17.6% compared to 18.2%, respectively [212]. In addition, metal porphyrins of Cu and Zn are also promising HTMs, as shown in Figure 8 [213]. CuP and ZnP showed suitable band alignment. Especially, ZnP-based PSCs exhibited a high PCE of 17.78%, which was comparable to that of bare spiro-OMeTAD-based PSCs (18.59%). Cao et al. (2018) investigated Co(II) and Co(III) porphyrin (CoP) compounds that could boost the PCE of PSCs to 19.6% [214]. Owing to their intrinsic high hole mobility, OMs are generally used as non-doped HTLs [207]. However, their strong absorption limits their use in conventional structures [94]. The use of organometallic materials and inorganic materials as HTLs provides a huge source for building solar cells that effectively convert light into electricity. However, there is still a gap from the experimental to the practical for which detailed factors should be optimized, such as the thickness, surface engineering, structure, cover of the cell, etc. Figure 9 shows some common HTL materials used in PSCs, including MO, MS, and MPc materials. Table 2 also provides a summary of the performance of solar cells employing inorganic and organometallic materials as HTLs, corresponding with the active materials used.

## 5. Conclusions and Outlook

In summary, LCSCs such as OSCs, PSCs, and DSSCs have been roughly developed and have reached significant improvements, achieving a PCE of over 24% or even higher with a tandem structure (a combination of 2–3 cells with different ranges of active light into a cell) or light concentrator facilities [217,218,219,220,221]. In order to obtain highly stable devices, the HTL layer has been fabulously investigated with a tremendous variety of materials, from inorganic to organic to organometallic compounds. In general, in comparison with IHTMs, OHTMs have better compatibility with organic active layer materials, which can decrease interfacial errors and increase charge transfer performance [14,17]. OHTMs also have lower costs and easier processability, which can enable large-scale fabrication of solar cells using methods such as printing or coating [14,17,81]. OHTMs have higher transparency and flexibility, which can enable the development of transparent and flexible solar cells for various applications [14,17,81]. In contrast, IHTMs still have higher stability under environmental conditions, such as heat, humidity, or oxygen. IHTMs have higher conductivity and lower resistance than OHTMS, which can improve the charge transport efficiency and decrease the series resistance of solar cells. IHTMs have a wider band gap than organic materials, which can improve the light-harvesting ability of solar cells. Lastly, IHTMs can be produced with simple methods, while OHTMs need multi-step synthesis [14]. The combination of these materials with organic materials balances their properties, in which the high performance of the OHTL and the stability of inorganic materials can be established from their compositions, such as GO/PEDOT:PSS, NiO_x_/PEDOT:PSS, or NiPc-doped spiro-OMeTAD [113,136,212]. Sulfur-based or organometallic materials show a low polarization surface that is easily compacted with the growth of perovskite, improving the performance of PSCs [196,214]. Even though many achievements have been recorded, practical application of these materials is still not well established due to the cost-effectiveness of the processing method, and optimizations need to be investigated. The variable wettability of different HTLs affects the morphology, contact angle, and stability [222]. The rough surfaces and surfaces with pinholes act as defects that trap charge carriers, thereby reducing the PCE. Some materials have high wettability and low contact angles, such as PTAA, spiro-OMeTAD, and PEDOT:PSS; however, surface treatment is also needed to obtain a high-quality surface. Meanwhile, P3HT wettability depends on its formulation and surface treatment. Intrinsic rGO or TMS exhibit high hydrophobicity and functionalization can help reduce the contact angle with another surface, reducing traps and improving the efficiency of the charge transfer process. Meanwhile the wettability of MO depends much on its structure. In general, UV-O treatment greatly helps the surface of inorganic HTLs. In addition, some techniques can be applied to inorganic HTLs, such as modifying thin casting films using the spray method, static/dynamic spin-coating method, using ionic liquids, or incorporating organic molecules as additives [223,224,225,226]. Recently, organometallic materials have become promising to replace traditional HTLs with lower costs and high performance. Therefore, there is still a lot of room for studying new materials or optimizing/combining the layer-by-layer structure. Hence, tandem cells are also promising to overcome the light absorption efficiency issues; therefore, the selection of different HTLs for each top/bottom cell needs to be performed. We believe this overview of HTLs could contribute to having a full, bright picture of flexible solar cells in the future. 

## Figures and Tables

**Table 1:** Summarization of organic hole transport materials.

HTMs	ABSORBER	V_OC_	J_SC_	FF	PCE	Ref.
**Spiro-OMeTAD**						
**Pristine Spiro-OMeTAD**	CH_3_NH_3_PbI_3_	1.0	18.30	73.64	13.48	[43]
**Mg-TFSI_2_-doped Spiro-OMeTAD**	Triple-cation perovskite (Cs_0.06_FA_0.79_MA_0.15_PbI_2.55_Br_0.75_)	1.091	22.62	76.70	18.93	[36]
**Ca-TFSI_2_-doped Spiro-OMeTAD**		1.074	22.54	76.50	18.69	
**Zn-TFSI_2_-doped Spiro-OMeTAD**	Triple-cation perovskite	1.162	23.78	78.80	22.00	[37]
**Spiro-OMeTAD(TFSI)_2_**	Rb_0.05_Cs_0.05_FA_0.8_MA_0.07_PbBr_0.4_I_2.57_	1.08	23.9	75.00	19.30	[38]
**PTAA**						
**Pristine PTAA**	CH_3_NH_3_PbI_3_	1.08	22.44	71.00	18.11	[61]
**UV-Treated dopant-free PTAA**	Cs_0.05_(MA_0.17_FA_0.83_)_0.95_Pb(I_0.83_Br_0.17_)_3_	1.08	22.74	78.00	19.17	[67]
**PTAA-MA**	Cs_0.05_(MA_0.17_FA_0.83_)_0.95_Pb(I_0.83_Br_0.17_)_3_	1.13	22.86	78.16	20.23	[57]
**PEDOT:PSS**						
**Solvent-treated PEDOT:PSS (ethylene glycol + methanol)**	MAPbI_3_	1.04	22.21	79.00	18.18	[69]
**PEDOT:PSS + PCDSA (MeOH treatment)**	MAPbI_3_	0.90	18.88	72.00	13.01	[71]
	PTB7-Th:PC_70_BM	0.72	17.40	59.00	7.71	
	P3HT:PC_60_BM	0.56	9.07	61.00	3.18	
**P3HT**						
**Pristine P3HT**	(FAPbI_3_)_0.95_(MAPbBr_3_)_0.05_ + wide-bandgap halide perovskite (WBH)	1.144	24.92	79.50	22.70	[33]
**P3HT-MDN**	Cs_0.05_FA_0.85_MA_0.10_Pb(Br_0.03_I_0.97_)_3_	1.15	24.58	75.02	22.87	[72]
	**Other OHTMs**					
**PCz1**	Cs_0.08_FA_0.80_MA_0.12_Pb(I_0.88_Br_0.12_)_3_	1.04	22.41	77.55	18.04	[79]
**Hydrogen bonding PCz**	Cs_0.05_(MA_0.17_FA_0.83_)_0.95_Pb(I_0.83_Br_0.17_)_3_	1.03	22.6	64.7	15.1	[80]
**Benzothiophene CDs**	MAPbI_3_	1.04	19.57	64.79	13.22	[81]
**RCP of BDT and BT**	CH_3_NH_3_PbI_3_	1.08	21.9	75	17.3	[82]
**BTT**	MA_0.7_ FA_0.3_PbI_3_	1.14	23.4	78.9	20.9	[19]
**BDD**		1.23	23.2	62.8	16.3	

**Table 2:** Summarization of inorganic and organometallic hole transport materials.

HTM	ABSORBER	V_OC_	J_SC_	FF	PCE	Ref.
**GO- and carbon derivative-based HTLs**						
**GO**	P3HT:PC_61_BM	0.57	11.40	54.30	3.50	[101]
**PEDOT:PSS/GO**	PTB7:PC_71_BM	0.76	16.42	65.80	8.21	[103]
**PEDOT:PSS/GO**	PCDTBT:PC_71_BM	0.82	10.44	50.00	4.28	[102]
**S-GO**	P3HT:PC_61_BM	0.61	10.15	71.00	4.37	[104]
**CL-GO**	PCDTBT:PC_71_BM	0.88	13.65	54.70	6.56	[105]
**rGO**	P3HT:PC_61_BM	0.58	9.87	65.00	3.71	[106]
**N-GO/SnO_2_**	Rb-doped-FA-MA-Br-mixed perovskite	1.17	18.87	74.93	16.54	[108]
**Cu@RGO**	MAPbI_3_	0.97	19.20	69.80	13.23	[109]
**F5-GO/PEDOT:PSS**	PTB7:PC_71_BM	0.78	15.31	63.00	7.52	[110]
**F-rGO**	PTB7:Th:PC_71_BM	0.78	16.89	64.80	8.6	[111]
**GQDs**	Dr3TBDT:PC_71_BM	0.92	11.36	65.20	6.82	[122]
**CNT/PEDOT:PSS**	P3HT:PC_61_BM	0.55	11.58	58.00	3.69	[123]
**O-MWCNTs**	Cs-FA-MAPbIBr	0.99	21.96	41.09	8.99	[215]
**Metal oxide HTLs**						
**NiO**	PDTG-TPD:PC_71_BM	0.82	13.90	68.40	7.82	[128]
**NiO**	MAPbI	0.98	21.10	78.00	16.1	[130]
**NiO**	CsFA-MA-Pb-I-Br	1.02	21.00	85.00	16.7	[131]
**Cu doped NiO**	MAPbI	1.10	21.73	75.30	18.02	[132]
**NiMgO_x_**	MAPbI	1.07	21.30	79.00	18.20	[133]
**NiO_x_**	FA-MA-Pb-I-Cl	1.12	23.70	76.00	20.20	[138]
**NiO**	AVA-MAPI	0.83	20.90	65.50	10.91	[134]
**S-NiO_x_**	RP(BDT-PDBT):PC_71_BM	0.71	9.85	63.00	4.45	[135]
**MoO_x_**	PM6:Y6:PC_71_BM	0.84	26.71	74.22	17.21	[147]
**MoO_x_**	PB3T2:IT-M	0.96	16.20	68.00	10.50	[150]
**s-MoO_x_**	P3HT:PC_61_BM	0.59	9.50	68.00	3.80	[149]
**WO_3_**	P3HT:PC_71_BM	0.61	12.80	60.40	4.80	[216]
**WO_x_-PEDOT:PSS**	MAPbICl	0.97	20.76	70.90	14.30	[155]
**Ti_3_C_2_T_x_/WO_3_/PEDOT:PSS**	MAPbI_3_	0.90	22.47	60.02	12.26	[153]
**O-CuO_x_**	PTB7:PC_71_BM	0.74	16.44	71.00	8.52	[164]
**Metal sulfide HTLs**						
**MoS_2_**	MAPbI_3_	0.95	20.70	72.30	14.2	[186]
**O-MoS_2_ QDs**	PTB7:Th:PC_71_BM	0.79	16.90	65.00	8.66	[191]
**MoS_2_ Ns**		0.63	12.50	53.20	4.18	
**MoS_2_**	PBDB-T-2F:Y6:PC_71_BM	0.81	25.30	71.00	14.9	[192]
**WS_2_**		0.83	26.00	72.00	15.6	
**CuS**	MAPbI_3_	1.02	22.30	71.20	16.2	[195]
**Cu_x_S-GO**	TiO_2_/CuInS_2_	0.60	16.06	62.90	6.07	[198]
**Cu_2_SnS_3_**	FAPbI_3_	1.04	24.14	64.92	16.33	[201]
**Organometallic HTLs**						
**Cop**	CsFAMAPbIBr	1.13	23.62	76.66	20.47	[214]
**Ni-Pc**	FAPbI_3_@MAPbBr_3_	0.895	18.50	63.80	10.6	[212]
**Ni-Pc/V_2_O_5_**		1.08	23.10	73.40	18.3	
**Zn-Pc**	MAPbIBr	1.02	22.36	71.43	16.23	[211]
**CuPc**	FAPbI_3_@MAPbBr_3_	1.07	20.52	66.30	16.36	[213]
**ZnPc**		~1.10	21.75	71.30	17.78	

## Data Availability

Not applicable.
